# Identification of a miRNAs signature as potential biomarker of mesenchymal phenotype in neuroblastoma patients

**DOI:** 10.1186/s40364-025-00866-z

**Published:** 2025-11-26

**Authors:** Silvia Lampis, Alessandro Paolini, Virginia Di Paolo, Angela Galardi, Salvatore Raieli, Evelina Miele, Lauriane Lemelle, Francesco Fabozzi, Annalisa Serra, Angela Mastronuzzi, Maria Antonietta De Ioris, Andrea Masotti, Franco Locatelli, Angela Di Giannatale

**Affiliations:** 1 Hematology/Oncology and Cell and Gene Therapy Unit, Bambino Gesù Children’s Hospital, IRCCS, Rome, Italy; 2 Multifactorial and Complex Phenotype Research Area, Bambino Gesù Children’s Hospital-IRCCS, Rome, Italy; 3 Microenviroment and Biomarkers in Solid Tumors Unit, Department of Experimental Oncology, Fondazione IRCCS, Istituto Nazionale dei Tumori, Milan, Italy; 4 Oncodesign SA, Dijon, 21079 France; 5 SIREDO Oncology Center (Care, Innovation and Research for Children and AYA with Cancer), PSL Research University, Institut Curie, Paris, France; 6 Department of Life Sciences and Public Health, Catholic University of the Sacraed Heart, Rome, Italy

## Abstract

Neuroblastoma (NB) is a heterogeneous tumor, ranging from cases with spontaneous regression (MS stage) to high risk (HR) tumors. Resistance to therapy presents a major challenge in the treatment and management of HR-NB, contributing to a poor prognosis. In these patients, the resistance to conventional treatment is also related to non-genetic features, such as the cellular plasticity. Novel studies have demonstrated the presence of two distinct cell phenotypes: adrenergic (ADRN) and mesenchymal (MES), which reflect the heterogeneity of NB. MicroRNAs (miRNAs) are small, endogenous and non-coding RNAs with the ability to regulate gene expression and may have a crucial role in controlling cell plasticity. However, the role of miRNAs in NB plasticity has not been investigated yet. We investigated miRNA signature in NB cells subtypes (MES and ADRN) and in the extracellular vesicles (EVs) released by them, to identify potential MES related biomarkers. Differentially expressed miRNAs were identified by RT-qPCR and subjected to gene ontology, KEGG pathway, and protein-protein interaction network analyses. Candidate miRNAs were validated in plasma-derived EVs from NB patients. We identified miR-199a-3p as strongly upregulated in the MES cells subtype. Moreover, its expression levels were significantly higher in primary cell lines derived from HR patients compared to low-risk (LR) ones. This was confirmed by a bioinformatics analysis in patient tissue obtained from TARGET NB dataset. Protein-protein interaction analysis uncovered a complex network, with FN1, CD44, and YAP1, identified as key genes upregulated by miR-199a-3p, all of which are closely associated with the MES phenotype. Among the miRNAs significantly upregulated in EVs derived from MES cell lines, miR-584a-5p was significantly higher in EVs isolated from plasma of HR and L/Intermediate(I)R patients compared to MS. MiR-584-5p is typically considered a tumor suppressor; to support this role miR-584a-3p resulted significantly upregulated in L/IR tumor tissue, both in the INSS and COG classifications (TARGET NB database). Our findings identified specific miRNAs as MES phenotype related biomarkers. Further studies should investigate the potential impact of miRNAs on plasticity-related pathways in order to open new therapeutic strategies.

**Supplementary Information:**

The online version contains supplementary material available at 10.1186/s40364-025-00866-z.

**To the Editor**,

Neuroblastoma (NB), the most common extracranial pediatric tumor, is classified into low (LR), intermediate (IR), and high risk (HR) groups, with HR-NB accounting for half of cases [1]. Resistance to therapy remains the major challenge in HR-NB treatment, with a 5-year survival rate of less 50% [2, 3]. HR-NB exhibits genetic features including *MYCN* amplification and chromosomal aberrations, however aggressiveness also arises from non-genetic factors. Recent studies identified two interchangeable phenotypes in NB, adrenergic (ADRN) and mesenchymal (MES), which display differential transcriptional and epigenetic profiles, with MES associated with aggressiveness and chemoresistance [4–7].

MicroRNAs (miRNAs), both free and carried into extracellular vesicles (EVs), regulate gene expression, and are known to modulate drug response in NB; however, their role in plasticity remains underexplored [8].

In this study, we investigate miRNA signatures in MES and ADRN NB subtypes, within the cells and in released EVs, aiming to identify potential MES-related biomarkers.

## miR-199a-3p is upregulated in mesenchymal neuroblastoma phenotype

**Fig. 1:**
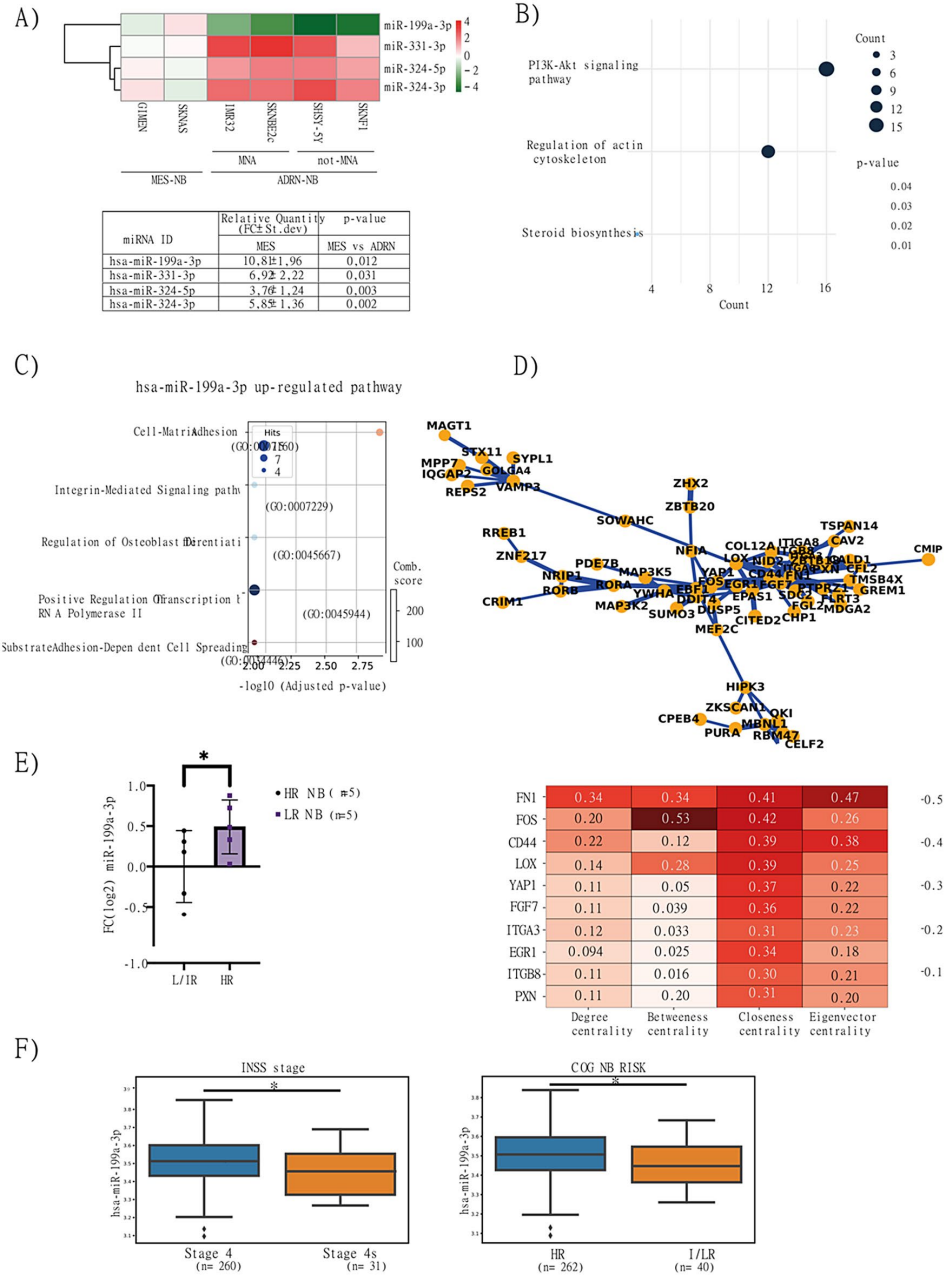
Characterization of dysregulated miRNAs in NB identifies miR-199a-3p linked to molecular pathways and patient risk. **(A)** Heatmap of the four statistically dysregulated miRNAs in ADRN cell lines, with a fold change (FC) 2 > FC > 2 compared to MES cell-lines. Upregulated and downregulated miRNAs are represented in red and green respectively; P-values are < 0.05. **(B)** Bioinformatics functional analysis of miR-199a-3p KEGG pathways. **(C)** Gene ontology analysis highlighting biological processes related to upregulated target genes of miR-199a-3p. **(D)** Protein-protein interaction (PPI) network derived from the STRING database for upregulated genes and a descriptive table of the hub genes accompanies PPI. **(E)** Expression levels of miR-199a-3p in NB primary cell lines, classified as HR (*n* = 5) and LR (*n* = 5). **(F)** Boxplot showing miR-199a-3p expression from the TARGET database, classified according to INSS and COG risk groups

NB cell lines were classified as MES (SKNAS, GIMEN) or ADRN (IMR32, SHSY5Y, SKNBe2C, SKNF1) based on gene expression profiles [5], (Figure S1A, S1B and Supplementary Table 1). The ADRN phenotype was further classified into *MYCN*-amplified (MNA) or not amplified (not-MNA). The miRNA analysis revealed dysregulation of nine miRNAs (Figure S1C). We then focused on statistically significant miRNAs that exhibited consistent expression patterns across all ADRN cells, ensuring a robust and meaningful comparison. Among those, miR-199a-3p was strongly upregulated in MES cells, while miR-324-5p, miR-324-3p, and miR-331-3p resulted downregulated (Fig. 1A).

## miR-199a-3p is functionally linked to mesenchymal-associated pathways

miR-199a-3p is computationally predicted to regulate 294 genes, including numerous components of key oncogenic pathways (Fig. 1B). We analyzed 262 h and 42 IR/ LR patients from the TARGET NB dataset and found that miR-199a-3p target genes were enriched in tumor from HR patients. Pathways upregulated by miR-199a-3p were primarily involved in cell adhesion, integrin signaling, and transcriptional regulation (Fig. 1C and Supplementary Table 2). Cross-referencing the list of miR199a-3p target in HR with STRING database we performed a protein-protein interaction analysis identifying a highly interconnected network involving FN1, CD44, and YAP1. Of note, these genes are known to be associated with the MES phenotype and poor prognosis (Fig. 1D and Supplementary Table 3).

## miR-199a-3p is upregulated in cell lines derived from HR patients

Primary cell lines derived from HR-tumor patients exhibited elevated miR-199a-3p expression compared to those from LR-tumor patients (*p* = 0.025), (Fig. 1E). Clinical characteristics are described in Supplementary Table 4. qPCR analysis in HR-lines showed expression of both phenotypes (MES and ADRN), with a higher MES phenotype in post-chemotherapy derived cell lines (Figure S1D). These findings support miR-199a-3p as a marker of aggressiveness and poor therapeutic response in NB. This was further validated in patient-derived datasets, where miR-199a-3p expression was significantly elevated in HR cases compared to IR/LR groups, according to both INSS and COG classifications (Fig. 1F).

## miR-199a-3p enhances aggressiveness of mesenchymal neuroblastoma by promoting proliferation and migration

To functionally validate the role of miR-199a-3p in NB aggressiveness, we modulated its expression in representative MES and ADRN NB cell lines and assessed the effects on cell proliferation and migration. Efficient modulation of miR-199a-3p levels was confirmed by quantitative real-time PCR (qRT-PCR) (Fig. S2A). Functional assays revealed that inhibition of miR-199a-3p in MES cell lines (GIMEN and SKNAS) resulted in a significant decrease in cellular proliferation compared to control-transfected cells. Conversely, overexpression of miR-199a-3p in ADRN cell lines (IMR32 and SH-SY5Y) led to a marked increase in proliferative capacity Fig. S2B). In parallel, Transwell migration assays demonstrated that miR-199a-3p downregulation significantly impaired the migratory potential of MES cells, whereas miR-199a-3p overexpression enhanced migration in ADRN cells. These findings were supported by representative immunofluorescence images (Fig. S3A) and quantitative analysis (Fig. S3B).

## Distinct miRNA profiles in EVs derived from MES and ADRN cells

**Fig. 2:**
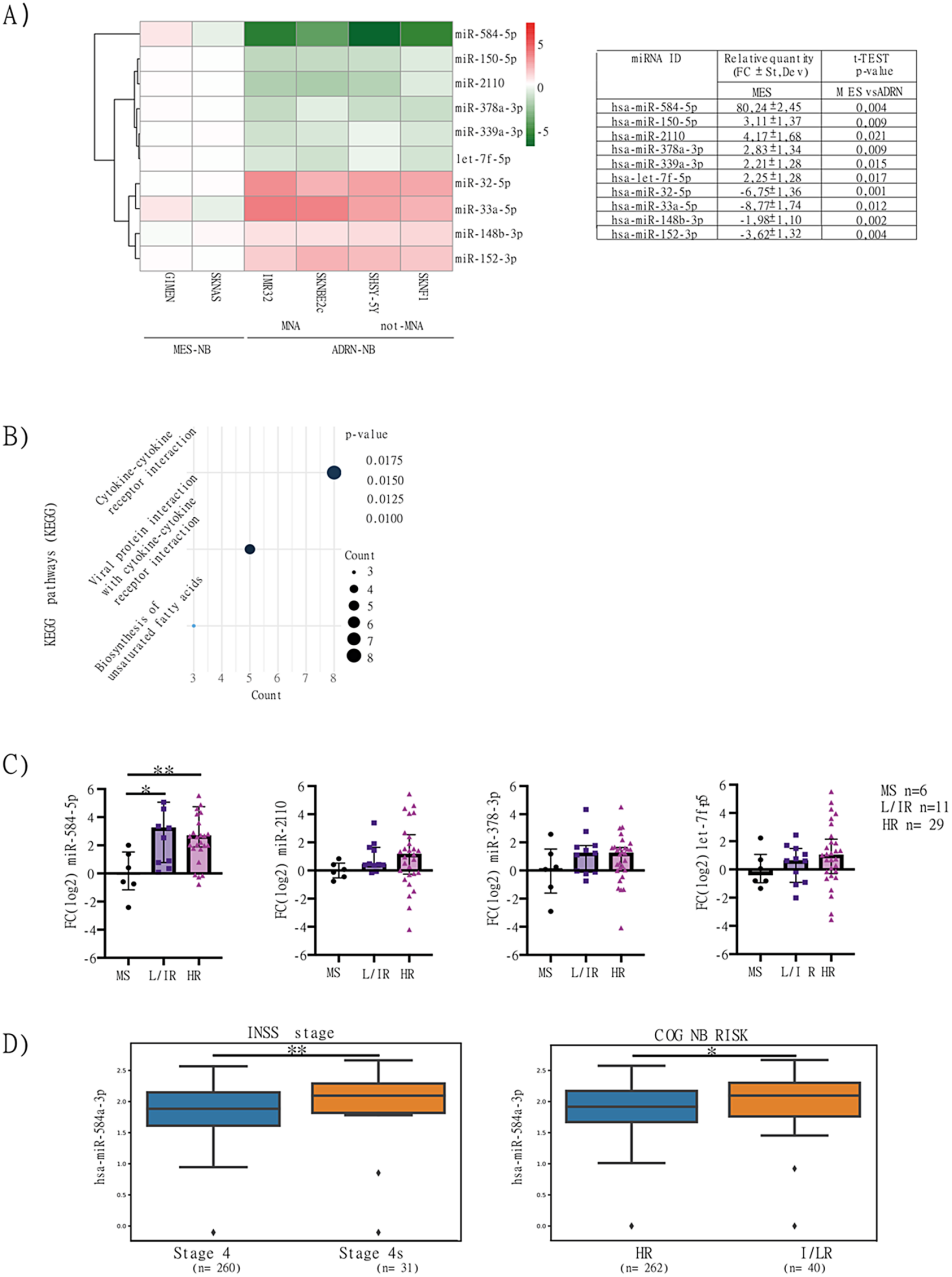
miRNA profiles in NB-derived EVs reflect patient pathobiology. **(A)** Heatmap of differentially expressed miRNAs in EVs derived from ADRN and MES NB cell lines. miRNAs with a fold change (FC) -2 > FC > 2 in ADRN-derived EVs compared to MES-derived EVs are shown. Upregulated and downregulated miRNAs are represented in red and green, respectively. P-values are (*P* < 0.05). **(B)** KEGG pathways enriched in miRNAs upregulated in MES phenotype. **(C)** Expression levels of miR-584a-5p, miR-2110, miR-378a-3p, and let-7f-5p Evs derived from plasma of NB patients classified as HR (*n* = 30), I/LR (*n* = 11), and MS stage (*n* = 6). **(D)** Boxplot showing miR-584a-5p expression from the TARGET database, classified according to INSS and COG risk groups. Statistical analysis was performed using Wilcoxon test (*P* < 0.05)

We next investigated miRNA cargo in EVs secreted by MES and ADRN cell lines, after EVs characterization following MISEV guidelines [9], (Figure S4 A-B-C). Ten miRNAs showed significant differential expressions. MiR-584-5p, miR-2110, miR-150-5p, and let-7f-5p were enriched in MES-derived EVs (Fig. 2A). Bioinformatic analysis revealed that target genes of MES EV-associated miRNAs were enriched in multiple signaling pathways, including cytokine-cytokine receptor interaction, viral protein-cytokine receptor binding, and biosynthesis of unsaturated fatty acids (Fig. 2B).

## Plasma EV miRNAs differentiate NB risk groups

To assess clinical relevance, we profiled plasma EV miRNAs from NB patients at diagnosis, including 6 MS (a unique subgroup often-experiencing spontaneous tumor regression), 11 L/IR and 29 h patients (Supplementary Table 5). We found that miR-584a-5p, typically considered as a tumor suppressor, was significantly downregulated in EVs from MS patients compared to those from L/IR (*p* = 0.036) and HR (*p* = 0.005 (Fig. 2C). TARGET dataset analysis revealed that miR-584a-3p was significantly upregulated in L/IR tumors (INSS *p* = 0.007; COG *p* = 0.037), (Fig. 2D). Pathway enrichment analysis revealed that miR-584a-5p target genes are involved in extracellular matrix organization and cell migration processes (Figure S4D).

## Discussion

Tumor heterogeneity drives cancer progression and affects drug response, hindering long-term remission. In NB, plasticity between MES and ADRN states influences treatment outcomes, impacting treatment response. MiRNAs regulate tumor plasticity and may promote differentiation and chemosensitivity, though their role in NB plasticity remains unexplored.

This study highlights miR-199a-3p as a biomarker of MES phenotype, being related with pathways associated with tumor plasticity, metastasis, and therapy resistance. MiR-199a-3p shows context-dependent roles in cancer [10] but acts as an oncogene in NB [11]. Our results indicate that miR-199a-3p may contribute to the aggressive behavior of MES NB cells by enhancing proliferation and migration. This suggests its potential as both a biomarker and a therapeutic target. Further investigation is necessary to elucidate its underlying mechanisms and validate its clinical relevance.

Additionally, we demonstrate that miR-584a-5p carried in plasma derived EVs may serve as potential biomarker of NB aggressiveness. The observed discrepancy - higher levels in EVs but reduced expression in aggressive tumors - supports its tumor-suppressive role.

By further elucidating the role of miRNAs in NB plasticity, our findings lay the groundwork for identifying novel biomarkers and therapeutic strategies aimed to improve outcomes for HR-NB patients.

## Supplementary Information

Below is the link to the electronic supplementary material.

Supplementary Material 1

Supplementary Material 2

Supplementary Material 3

Supplementary Material 4

Supplementary Material 5

Supplementary Material 6

## Data Availability

All data generated or analyzed during this study are included in this published article. Datasets are described in the material and methods section and are public available.

## Abbreviations

NB: Neuroblastoma
LR: Low Risk
IR: Intermediate Risk
HR: High Risk
ADRN: Adrenergic
MES: Mesenchymal
miRNA: MicroRNA
mRNA: Messenger RNA
EVs: Extracellular Vesicles
NTA: Nanoparticle Tracking Analysis
MISEV: Minimal Information for Studies of Extracellular Vesicles
RT: Reverse Transcription
GAPDH: Gglyceraldehyde-3-phosphate dehydrogenase
FC: Fold Change
PPI: Protein-protein interactions
BCA: Bicinchoninic acid
FACS: Flow cytometry
EMT: Epithelial-mesenchymal transition
