# Supplementary material for: Identification of a miRNAs signature as potential biomarker of mesenchymal phenotype in neuroblastoma patients
Source: Biomark Res. 2025 Nov 26;13:152. doi: 10.1186/s40364-025-00866-z (PMC12659618; doi:10.1186/s40364-025-00866-z)
Supplement: Supplementary file 1 — Supplementary Material 1 [file 40364_2025_866_MOESM1_ESM.docx]

**SUPPLEMENTAL METHODS**

**Cell line and cell culture**

Commercially available human NB cell lines, including IMR-32, SK-N-Be2C, SK-N-AS, and SK-N-F1, were purchased from ATCC, while GI-ME-N from DSMZ.

GI-ME-N, SK-N-AS and SH-SY-5Y were grown in DMEM Low-glucose (Euroclone Spa, Pero, MI, Italy); IMR-32 cells were maintained in IMDM medium (Euroclone Spa, Pero, MI, Italy); SK-N-Be(2)-c and SK-N-F1 cells were cultured in RPMI 1640 medium (Euroclone Spa, Pero, MI, Italy). All culture media were supplemented with 10% fetal bovine serum (FBS, Gibco, USA) previously depleted of bovine EVs by ultracentrifugation at 100 000 × g for 70 min, 2 mmol/L L-glutamine (Euroclone Spa, Pero, MI, Italy) and 100 g/mL penicillin- streptomycin (Euroclone Spa, Pero, MI, Italy). All cell lines were stored in liquid nitrogen and kept in culture at 37°C in a 5% (v/v) CO2 humidified incubator. Cell cultures were routinely tested and verified as mycoplasma-free.

**Isolation and characterization of EVs from cell lines**

EVs were purified from supernatants after 3-4 days of culture when cells had reached about 80% of confluence in T-75 flask, by sequential centrifugation. In detail, harvested supernatants were centrifuged at 500 x *g* for 10 min to remove any cell contamination, then spun at 12,000 x *g* for 20 min to remove any possible apoptotic bodies and large cell debris. Finally, EVs were collected by spinning at 100,000 x *g* for 70 min, twice, using a 70 Ti rotor (Beckman Coulter, Fullerton, CA, USA). The final pellet was resuspended in PBS and protein concentration was measured by BCA (Pierce, Thermo Fisher Scientific, Waltham, MA, USA) method with Sinergy H1 microplate reader (Biotek) against a standard curve of BSA. Two different biological replicates were prepared for each cell line. EVs preparations of IMR32, SHSY-5Y, SKNBE(2)c, GIMEN, SKNAS and SKNF1 were characterized following the MISEV 2023 guideline [9] by Western Blot (WB) and Nanoparticle Tracking Analysis (NTA) as previously described [16], (See Supplementary Methods).

**Patients and sample collection**

Ten primary tumor tissues derived from patients affected by NB undergoing needle biopsy or surgery at the Haematology/Oncology, Cell Therapy, Gene Therapies and Hemopoietic Transplant Department (IRCCS Bambino Gesù Children’s Hospital) were included in this study. Seven patients were enrolled at diagnosis and three post-treatment. Clinical characteristics of the above-mentioned patients are shown in **Supplementary Table 4**. Tissues were processed immediately after surgery, minced into 1–2 mm fragments using a sterile scalpel and resuspended in Advanced DMEM/F12 medium supplemented with 5% fetal bovine serum (FBS, Gibco, USA), 4 mmol/L L-glutamine (Euroclone, Italy), and 100 µg/mL penicillin-streptomycin (Euroclone, Italy). Primary cell lines were cultured at 37°C in a humidified atmosphere with 5% CO2. Once cells reached confluence, they were passaged and phenotypically characterized at early passages. (See Supplementary Methods).

Plasma samples at diagnosis were collected from 45 NB patients diagnosed at Haematology/Oncology, Cell Therapy, Gene Therapies and Hemopoietic Transplant. Among them 29 patients were diagnosed with HR, 3 IR, 8 LR and 6 with MS disease. The cohort consisted of 21 females and 24 males, with a median age of 34 months (range: 2–197 months).

Clinical characteristics of the above-mentioned patients are shown in **Supplementary Table 5**.

Written informed consent was signed by all parents and the study was approved by our Institutional Ethics Committee (protocol number IG2021_3111).

Whole blood was collected in EDTA tubes (BD Vacutainer, Reading, UK) and processed within 2 h. The samples were first centrifuged at 500×g for 10 min, and then supernatants were collected and centrifuged at 3000×g and then at 12,000×g for 20 min each. All the centrifugation steps were performed at 4 degrees. The plasma was collected and stored at -80°C until EVs isolation.

**Isolation and characterization of EVs from plasma**

Plasma EVs isolation and characterization was conducted as previously performed in “Colletti 2020 JEV” [17]. Isolation was performed using the commercial kit miRCURY™ Exosome isolation kit-serum and plasma (Qiagen) according to the manufacturer’s protocol. Briefly, 3 UI of Thrombin was added to 0.6 ml of plasma and incubated for 5 min at room temperature and centrifuged for 5 min at 10,000 × g. An amount of 0.5 ml of supernatant was collected, 200 μl of precipitation buffer A was added, resuspended by vortex for 5 s to mix and incubated for 60 min at 4 °C. After incubation, samples were centrifuged for 5 min at 500 g at RT and the supernatants were removed and discarded. Pellets were re-suspended by vortex in 270 μl resuspension buffer. The isolated EVs were characterized following the recommendations of “Minimal Information for Studies of Extracellular Vesicles” (MISEV) 2023 guideline [9]. The purified EVs samples were then processed for RNA extraction.

**RNA-extraction, mRNA and miRNA expression**

Total RNA for gene expression was extracted from cell lines using Total RNA Purification kit (Norgen Biotek Corp., Thorold, Canada). Reverse transcription (RT) was performed using the Improm-II Reverse Transcription System (Promega, Madison, WI, USA). The relative gene expression levels were measured by real-time -qPCR. TaqMan assays (Applied Biosystems, Life Technologies, Carlsbad, CA, USA) for ASCL1 (Hs00269932_m1), DBH (Hs01089840_m1), DLK1 (Hs00171584_m1), GATA2 (Hs00231119_m1), GATA3 (Hs00231122_m1), HAND1 (Hs02330376_m1), HAND2 (Hs00232769_m1), PHOX2a (Hs00605931_mH), PHOX2B (Hs00243679_m1), FN1 (Hs01549976_m1), SNAI2 (Hs00161904_m1), VIM (Hs00958111_m1) , YAP (Hs00371735_m1) and PRRX1 (Hs00246567_m1) were used. Samples were normalized according to the glyceraldehyde-3-phosphate dehydrogenase (GAPDH) mRNA (Hs99999905_m1) levels.

RNA for miRNA expression was isolated from cell lines and EVs isolated from cell supernatants and plasma using Total RNA Purification kit for cell lines and Plasma/Serum Circulating and Exosomal RNA Purification Mini Kit (slurry format) (Norgen Biotek Corp., Thorold, Canada) for EVs, following the manufacturer’s instructions. For extraction efficiency evaluation, spike-ins (RNA spike-in kit, Qiagen) were added to lysis buffer before plasma extraction. Extracted samples were stored at -80°C until further use. RT for miRNAs was performed mixing each RNA sample (2 μl) with two artificial RNAs (RNA spike-ins) used as RT controls, and the mixture (10 μl) was reverse transcribed at 42°C for 60 min using the miRCURY LNA™ Universal RTcDNA Synthesis Kit (Qiagen) following manufacturer’s instruction. The expression of each miRNA was evaluated by the Serum/Plasma Focus microRNA PCR panel (Qiagen) by QuantStudio 12K Flex Real-Time PCR System (Thermo Fisher Scientific, Waltham, MA, USA). The amplification curves were filtered (Ct<36), imported into the GenEx software (ver.6, Exiqon) and normalized by global mean. The mean of individual Cq values for the groups (NB cells and EVs) was considered to calculate the expression level (fold change [FC]).

**Bioinformatics Target Prediction of Dysregulated miRNAs and Gene Ontology**

We employed miR-prediction Database TargetScan to obtain validated and/or score predicted targets of miRNA significantly dysregulated in NB MES-type cells compared to NB ADRN cells. (<https://www.targetscan.org/vert_80/>). To identify the biological processes and signaling pathways associated with the predicted targets of the dysregulated miRNAs KEGG pathway enrichment analysis and annotation were performed by DAVID bioinformatics tool.

**Datasets used**

TARGET miRNA and TARGET gene expression profiles (RNAseq) used in this study were downloaded from TARGET data portal (https://ocg.cancer.gov/programs/target; data freely accessible). The differential expressed genes between HR, IR and LR patients from TARGET datasets were obtained using the limma package algorithm. The clinical information’s are available in the dataset and in the original data source. Plots were obtained using the Python Seaborn library.

**Pathway enrichment**

Pathway enrichment was conducted in Python (version 3.10) using GseaPy as the library and Go Biological Processes (version 2023) as the pathway base. Two different databases (miRNA BD and TargetScan) were used. The list of regulated genes for each pathway was obtained from the two databases, and the top 100 genes were chosen. Pathway enrichment was then conducted, and the top 5 pathways were plotted.

**Network analysis**

For TARGET patients, differential gene expression was conducted between HR and LR (as indicated in the clinical metadata). Subsequently, they were cross-referenced with data obtained from miRNA regulatory databases (miRNA BD and TargetScan). Genes that are most over-expressed (HR versus LR) and are simultaneously targets of a miRNA were selected. Pathway analysis was conducted on this list of genes using GseaPy. Protein-protein interactions (PPI) were obtained from STRING database (only those with a combined score above 400 were considered). The genes in the list were then plotted as nodes, and the interactions between them as edges. Graph analysis was conducted in Python using the NetworkX library. The biological graph was compared with a random graph of equal size according to the Erdős-Rényi model. Classical measures of centrality were used (**Supplementary Table 3**).

**Supplementary methods**

**Extracellular Vesicles Characterization (NTA, WB)**

EV preparations were verified following the recommendations of MISEV2023 [9] The NS500 nanoparticle characterization system (NanoSight) equipped with a blue laser (405 nm) was used to characterize EVs size and particle number.

Protein extraction was performed with Cell lysis buffer (Cell Lysis Buffer (10X) #9803 Cell Signalling Technology) containing 10 mM phenylmethylsulphonyl fluoride (PMSF #93482 Sigma) as a protease inhibitor.

Lysates were incubated on ice for 30 min and centrifuged at 12 000 × g for 20 min at 4°C. Equal micrograms (10 μg) of proteins quantified with bicinchoninic acid (BCA) assay (Thermo Scientific) and boiled in SDS sample buffer (4x Laemmli Sample Buffer BIORAD cat. #161-0747) were resolved on 10% SDS-PAGE and transferred to PVDF membranes (Immobilon®-P Transfer Membrane Merck Millipore cat. IPVH00010). Blots were blocked for 1 h in PBS-T (PBS plus 0.05% Tween-20), 5% non-fat, dried milk and probed overnight at 4°C with anti-TSG101 (4A10) ab83 (Abcam), anti-CD9 (C-4) sc-13118 (Santa Cruz Biotechnology), anti-CALNEXIN (E-10) sc-46669 (Santa Cruz Biotechnology), and anti-HSP90α/β (F-8) sc-13119 (Santa Cruz Biotechnology). Immunocomplexes were detected with horseradish peroxidase-conjugated species-specific secondary antibodies (Santa Cruz Biotechnology) followed by enhanced chemiluminescence reaction with Immobilon Western Chemiluminescence HRP substrate WBKLS0100 (Millipore).

**Modulation of miR-199a-3p Expression in Neuroblastoma Cell Lines**

Cells were transiently transfected with mirVana™ miRNA inhibitor or mimic targeting hsa-miR-199a-3p (Invitrogen, Thermo Fisher Scientific) using Lipofectamine™ RNAiMAX (Invitrogen) according to the manufacturer’s instructions. Transfections were performed in Opti-MEM reduced-serum medium (Thermo Fisher Scientific) at a final concentration of 30 pmol per well. MES cells (GIMEN and SKNAS) were transfected with the miRNA inhibitor, whereas ADRN cells (IMR32 and SH-SY5Y) were transfected with the miRNA mimic.

**Cell proliferation assay**  Cell proliferation was assessed using the Cell Counting Kit-8 (Sigma Aldrich) according to the manufacturer’s instructions. Briefly, cells were seeded in 96-well plates at a density of 3 × 10^3 cells/well in triplicate biological replicates per condition. After transfection, cell viability was measured at 24, 48, and 72 hours by adding 10 µL of CCK-8 reagent to each well and incubating for 2 hours at 37°C. Absorbance was recorded at 450 nm using a microplate reader (Synergy h1 microplate reader, Agilent BioTek). Data were normalized to the 0-hour time point of the control.

**Migration assay** Migration assays were performed using Transwell chambers with 8 µm pore size polycarbonate membranes (Corning, USA). Cells were transfected for 48 hours, then harvested and resuspended in serum-free medium. A total of 3 × 10^4 cells in 300 µL serum-free medium were added to the upper chamber, while 1000 µL of complete medium was placed in the lower chamber as a chemoattractant. After 24 hours incubation at 37°C, non-migrated cells on the upper surface were removed with a cotton swab. Migrated cells were fixed in 4% paraformaldehyde, stained with DAPI (1 mg/mL, Sigma), and counted in five randomly selected fields per filter using ImageJ software (NIH).
