# Supplementary figures and images for: Identification of a miRNAs signature as potential biomarker of mesenchymal phenotype in neuroblastoma patients
Source: Biomark Res. 2025 Nov 26;13:152. doi: 10.1186/s40364-025-00866-z (PMC12659618; doi:10.1186/s40364-025-00866-z)

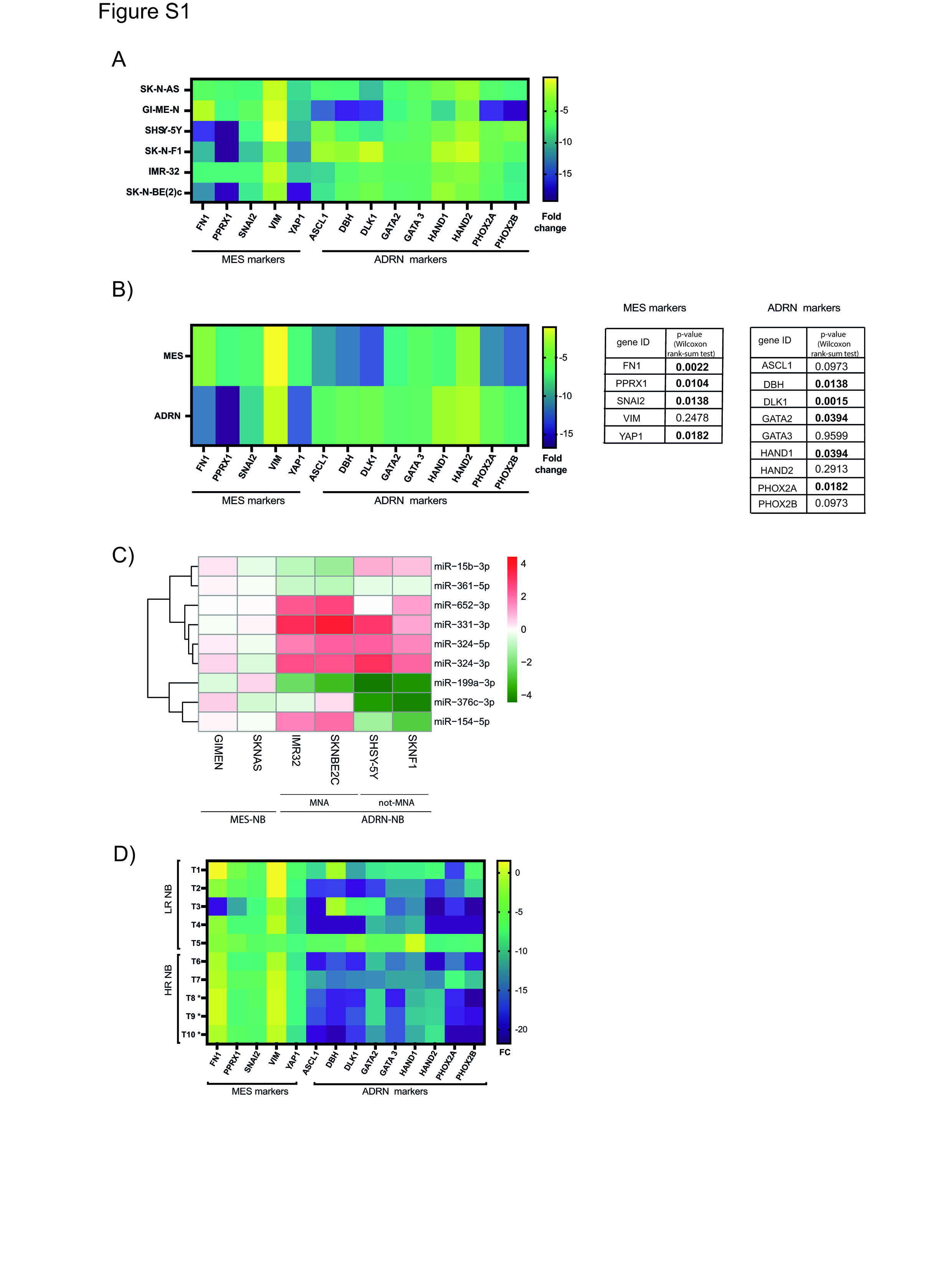

Supplement: Supplementary file 2 — Supplementary Material 2 [file 40364_2025_866_MOESM2_ESM.tif]
